# Epigenetic analyses suggest different pathways during pregnancy for development of type 1 diabetes in children with high versus low‐neutral human leukocyte antigen‐risk

**DOI:** 10.1111/joim.70077

**Published:** 2026-02-25

**Authors:** Shamila D. Alipoor, Angelica Ahrens, Julia Åkesson, Thomas Hillerton, Mika Gustafsson, Maria Lerm, Johnny Ludvigsson

**Affiliations:** ^1^ Division of Pediatrics, Department of Biomedical and Clinical Sciences Linköping University Linköping Sweden; ^2^ Department of Biomedical and Clinical Sciences (BKV) Linköping University Linköping Sweden; ^3^ Microbiology and Cell Science Department University of Florida Gainesville Florida USA; ^4^ Division of Bioinformatics, Department of Physics, Chemistry and Biology Linköping University Linköping Sweden; ^5^ PredictMe AB Linköping Sweden; ^6^ Crown Princess Victoria Children's Hospital, Linköping University Hospital Linköping Sweden

**Keywords:** autoimmune diseases, epigenomics, human leukocyte antigen (HLA) antigens, Type 1 diabetes mellitus (T1D), Type 2 diabetes mellitus (T2D)

## Abstract

**Background and objective:**

The development of Type 1 diabetes (T1D) is shaped by genetic predisposition and epigenetic regulation. Human leukocyte antigen (HLA) risk alleles are major genetic determinants, but the epigenetic landscape in relation to disease onset remains unclear. Early‐life epigenetic modifications may reveal how environmental and epigenetic factors interact in T1D pathogenesis.

**Methods:**

We investigated epigenetic differences in cord blood DNA from individuals with different HLA risk alleles who later developed T1D using epigenome‐wide association studies.

**Result:**

High‐risk HLA carriers showed differentially methylated genes (DMGs) mainly involved in immune and autoimmune processes, resembling patterns in other autoimmune diseases. In contrast, low‐to‐neutral risk carriers exhibited DMGs linked to signaling cascades, metabolic pathways, and Type 2 diabetes–related mechanisms such as beta cell function and insulin signaling.

**Conclusion:**

These findings indicate that heterogeneity in T1D pathogenetic mechanisms based on HLA background may influence disease development.

## Graphical abstract

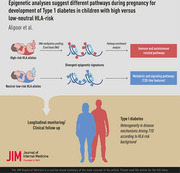

**Abbreviations**
ABIS: All Babies in Southeast Sweden
Daisy: Diabetes Autoimmunity Study in the Young
DIP: diabetes in pregnancy
DIPP: Type 1 diabetes prediction and prevention study
DMCs: differentially methylated CpG sites
DMGs: the genes associated with CpG sites
HR: high risk
LR: low‐to‐neutral risk
NT1D: other autoimmune diseases, excluding T1D
T1D: Type 1 diabetes
T2D: Type 2 diabetes
TEDDY: The Environmental Determinants of Diabetes in the Young TEDDY study

## Background

Type 1 diabetes (T1D) is characterized by destruction of insulin‐producing beta cells in the pancreas, leading to the deficiency of insulin and lifelong reliance on external insulin therapy. The etiology of T1D is still unknown, even if there are many good hypotheses [1].

So far, nearly 50 genetic loci have been shown to be associated with T1D susceptibility. The most critical genetic factor remains the human leukocyte antigen (HLA) region, located on chromosome 6 [2]. This region is highly variable among individuals, and specific combinations of HLA Class II alleles considerably influence disease risk. These genetic differences modulate the immune response by altering antigen presentation, playing a key role in the autoimmune processes that target pancreatic beta cells in T1D [2].

Although genetic predisposition is a key factor in T1D risk, there is a dynamic interaction between environmental influences and genetic susceptibility, which plays an important role in disease onset and progression. Emerging evidence highlights the central role of epigenetic mechanisms that link environmental factors to genetic risk, offering new insights into the complex etiology of T1D [3]. Epigenetic changes, including DNA methylation and histone modification, can stably alter gene expression and are inherited during cell division. DNA methylation occurs at cytosine residues mainly in the context of CpG dinucleotides and is associated with transcriptional silencing, which may function as a mediator in response to environmental stimuli, contributing to disease development through its influence on gene expression [4, 5]. It is known that epigenetic patterns are associated with the development of T1D [6, 7, 8] as well as other autoimmune diseases [6], whereas it is thought that the related epigenetic changes mainly appear after birth [9]. Although some studies report similar methylation profiles between T1D patients and healthy individuals [10], others find differences in methylation and histone marks at key HLA loci, suggesting that these epigenetic changes contribute to disease susceptibility and progression [11, 12]. For example, differential methylation of *HLA‐DR* and *HLA‐DQ* genes, central to antigen presentation and immune response, has been observed in T1D patients with high‐risk (HR) HLA alleles [11]. Changes in histone acetylation and methylation at *HLA‐DRB1* and *HLA‐DQB1* promoters further alter chromatin structure and gene expression, promoting autoimmunity [12].

Despite the recognized importance of HLA risk alleles and their contribution to T1D disease development, the mechanisms underlying T1D development in individuals with protective HLA alleles remain poorly understood.

Most cohorts, such as TEDDY [13], Daisy [14], DIPP [15], and BabyDiab [16], follow only individuals with high genetic risk, which may overshadow environmental factors and make it difficult to find such factors and get deeper knowledge in pathogenetic mechanisms. The All Babies in Southeast Sweden (ABIS) [17, 18] is a birth cohort including a general population, which means that we are able to study why individuals with no or low genetic HLA risk still develop T1D. We have previously found differences in gut microbiome associated to HLA‐risk [19, 20].

In this study, using cord blood DNA from the ABIS cohort, we conducted systematic epigenome‐wide association studies (EWAS) to assess DNA methylation profiles in individuals with LR or HR HLA alleles who later developed T1D and compare these with profiles observed in other autoimmune diseases in order to determine whether early‐life epigenetic differences exist among individuals with varying HLA risk alleles who go on to develop T1D.

## Study design and characteristics

ABIS cohort [17, 18] follows, to the present day, 17,055 individuals born between October 1997 and 1999 in Southeastern Sweden. Families were recruited from nine obstetric clinics across all hospitals in the counties of Östergötland, Småland, Blekinge, and Öland. They received oral and written information and were also offered video information and were then asked to join the study. Out of the 21,700 children born in the region, 78.6% of families provided informed consent for their child to participate. Parents completed extensive questionnaires and diaries from the child's birth through >20 years of age, with ABIS children themselves answering questionnaires at later visits. Cord blood samples were collected from all ABIS children. The ABIS register is connected to the Swedish National Patient Register, giving diagnosis of diseases, and to the National Drug Prescription Register, which has been used for validation of diagnosis of T1D.

Four groups of individuals were included in this study: (1) Individuals with HR HLA alleles who later developed T1D (T1D‐HR) (*n* = 25), (2) Individuals with low‐neutral risk HLA alleles who later developed T1D (T1D‐LR) (*n* = 17), (3) Individuals with HR HLA alleles who developed other autoimmune diseases, excluding T1D (NT1D‐HR) (*n* = 25), and (4) healthy individuals with neutral or low‐neutral risk HLA alleles (*n* = 39).

Autoantibody profiling at diagnosis showed that 75% of participants were positive for at least one islet autoantibody (GADA, IA‐2A, IAA, or ZnT8), and the participants were diagnosed at a median age of 11.4 years.

### Ethical statement

Ethical approval for ABIS was obtained by the Research Ethics Committees of Faculty of Health Science at Linköping Univ., Ref. 1997/96287 and 2003/03‐092 and the Medical Faculty of Lund University Dnr 99227, Dnr 99321. Connection to National Registers was approved (Dnr 03‐513 and 2018‐318‐32).

### DNA extraction

Genomic DNA was isolated from whole blood using the QIAamp DNA Mini Kit on the QIAcube Connect automated platform (Qiagen, Germany). DNA concentration was measured with the Quantus Fluorometer and QuantiFluor ONE dsDNA System (E4871, Promega, USA), and DNA quality was evaluated using a NanoDrop ND‐1000 spectrophotometer (Thermo Fisher Scientific, USA).

### Methylation array data

Genomic DNA (250 ng/sample) samples were bisulfite‐converted using EZ DNA Methylation Kit (Zymo Research) and profiled using the Illumina MethylationEPIC v1.0 array. BeadChips were scanned on an Illumina NextSeq 550.

### Data preprocessing and quality control

Methylation signal intensities were extracted from raw IDAT files using the minfi R package (v1.50.0) in R version 4.4.3 [21, 22]. During the initial preprocessing stage, signal intensities were normalized and converted to β‐values using the intra‐sample beta‐mixture quantile (BMIQ) normalization procedure [23] and the inter‐array Gaussian mixture quantile normalization (GMQN) method [24] implemented in the GMQN R package (v0.1.0) [24].

Probe‐level quality control and filtering were applied in the subsequent steps. Probes with missing values in more than 25% of the samples were excluded. Additionally, cross‐reactive probes, non‐CpG probes, probes mapping to known single nucleotide polymorphisms (SNPs), and probes located on sex chromosomes (X and Y) were discarded using the ChAMP R package (v2.34.0) [22]. Remaining missing values were imputed using *k*‐nearest neighbor imputation via the scikit‐learn Python package [25].

To identify and correct technical artifacts, singular value decomposition (SVD) analysis was performed using a modified version of the SVD function from the ChAMP R package. Major sources of variation significantly associated (*p* < 0.05) with SVD components explaining >3% of the variance, such as slide and array effects, were adjusted using the ComBat algorithm from the sva R package (v3.52.0) [26]. A follow‐up SVD analysis confirmed successful artifact removal.

Finally, data quality was comprehensively evaluated using density plots of β‐value distributions. Principal component analysis was performed to visualize sample clustering and confirm preprocessing efficiency.

### Cellular composition analysis

To estimate cell type composition in each sample, we used the *EpiDISH* R package (v2.20.1) [27], applying the robust partial correlations (RPC) method with the “cent12CT.m” whole blood reference panel for the EPIC array. This reference enables deconvolution of 12 blood cell subtypes, including NK cells, monocytes, Treg cells, basophils, and both memory and naive subsets of B cells, CD4+ T cells, and CD8+ T cells [28]. Differences in cell type proportions were evaluated using Kruskal–Wallis *H*‐test.

### Differential DNA methylation analysis

Differentially methylated CpGs (DMCs) between groups were identified using the *limma* R package (v3.60.6) [29]. For each CpG, linear models were fitted adjusting for sex and cell type proportions that differed significantly between groups (*p* < 0.05). Empirical Bayes moderation was applied to stabilize variance estimation, and differential methylation was assessed using moderated *t*‐statistics. We applied a *p* value cutoff of 0.05 and a |log FC| cutoff of 0.05 to determine whether a CpG was differentially methylated [30, 31]. The identified DMCs were mapped to corresponding differentially methylated genes (DMGs) and genomic regions using Illumina EPIC array annotations *ilm10b4.hg19* [32]. The results were visualized as heatmaps using *ComplexHeatmap* package (v2.10.0) [30, 31] and as volcano plots using *ggplot2* [33] in R (v3.4.4).

### Pathway enrichment analysis and network analysis

The identified DMCs were analyzed using *EnrichR* [34] and the Kyoto Encyclopedia of Genes and Genomes pathway database [35]. To enhance visualization and interpretation of enrichment results, a dot plot was generated using the *ggplot2* R package. Gene intersections across pathways were visualized with the *enrichplot R* package [36, 37].

DMC‐associated genes were integrated into a protein–protein association network from the STRING database (v12.0) [38], and functional modules were identified using a consensus approach combining the DIAMOnD [39] and CliqueSum [40] inference methods. Module enrichment for gene ontology (GO) [41] biological processes was subsequently assessed.

## Statistics

Differences in cell type proportions were tested using the Kruskal–Wallis *H*‐test, with significance defined as *p* < 0.05. Differential DNA methylation was evaluated with limma using moderated *t*‐statistics, and CpG sites were considered differentially methylated at *p* < 0.05 and |log FC| > 0.05. For pathway enrichment, significance was set at *p* < 0.05.

## Results

### DNA methylation patterns in cord blood of T1D individuals vary by HLA risk

**Comparison of epigenetic patterns in T1D developers with HR versus LR HLA alleles** as a quality control step; we first performed a comprehensive quality assessment, which confirmed high data quality across all methylation profiles (Fig. S1). We then analyzed DNA methylation in cord blood from individuals carrying either HR or LR HLA alleles who later developed T1D. Most of the detected DMGs were hypermethylated in HR carriers (67%), whereas the remaining 33% exhibited a hypomethylated pattern in this group. The genomic distribution of associated CpG sites is detailed in Table S1, with most CpGs located within gene bodies, followed by the region 200–1500 kb upstream of the transcriptional start site (Fig. S2).

In the HR group, distinct methylation patterns were observed among HLA genes as well as the genes with critical role in immune reactions. Specifically, *HLA‐DQA1* and *HLA‐DQA2* were hypomethylated in this group compared to LR HLA carriers.

**Fig. 1:**
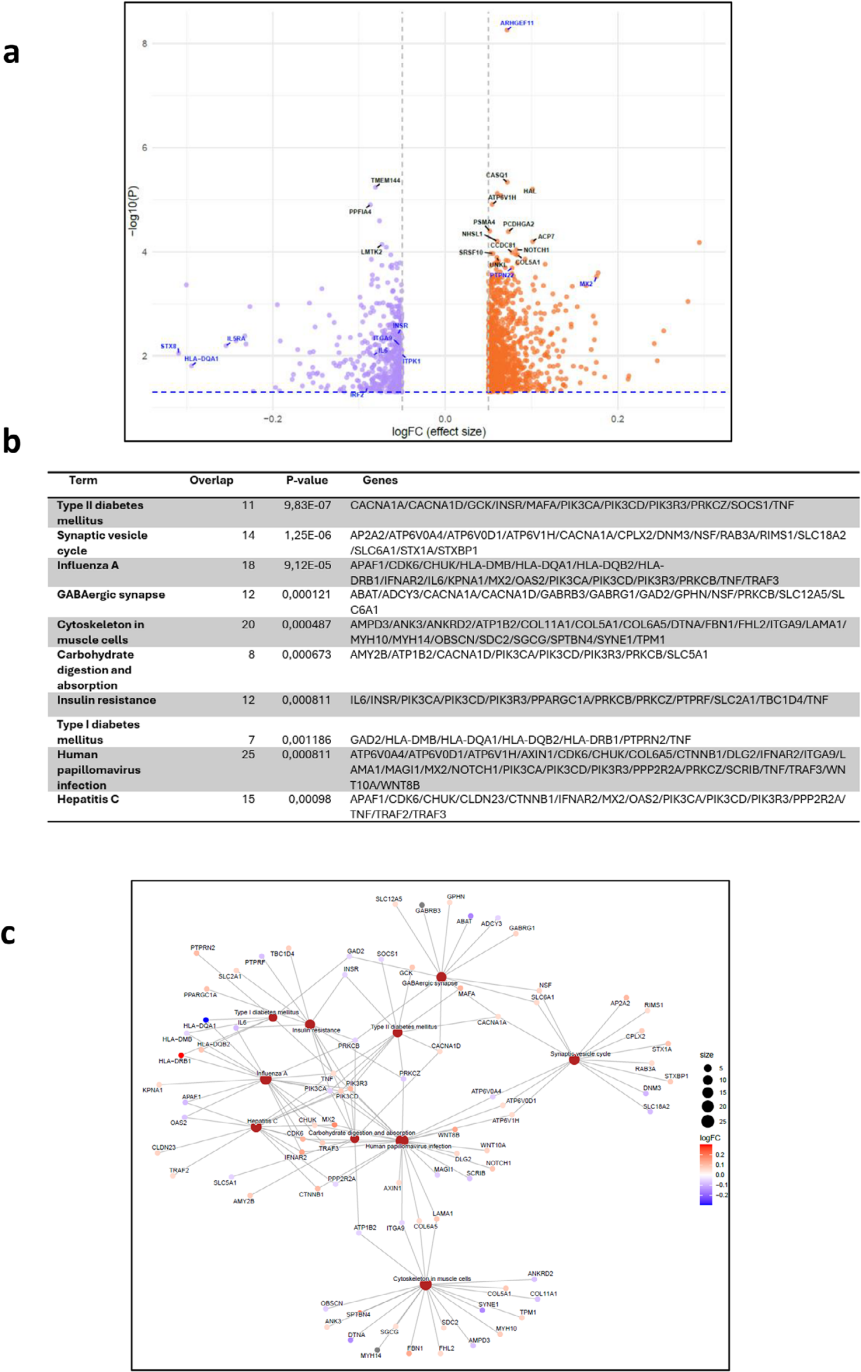
Methylation analysis comparing individuals with T1D carrying HR human leukocyte antigen (HLA) alleles to those with LR alleles. (a) Volcano plot illustrating the DMGs between the two groups (log FC: blue: <−0.05, red: >0.05). (b) Kyoto Encyclopedia of Genes and Genomes (KEGG) pathway enrichment analysis of DMGs (Top 10 pathways enriched from the DMGs, p < 0.05). (c) Network visualization of enriched pathways and associated genes from DMC analysis. The network visualizes the relationships between enriched biological pathways and their associated genes. Each colored node (circle) represents either a pathway (in red) or a gene, with the gene nodes color‐coded according to their log fold change (log FC) values. The log FC scale indicates methylation levels. Edges (lines) indicate which genes are involved in which pathways, illustrating that individual genes can contribute to multiple biological processes. DMGs, differentially methylated genes; HR, high‐risk; LR, low risk; T1D, Type 1 diabetes.

Furthermore, in HR carriers, *ARHGEF11*, *PTPN22*, *PK13CD*, and *MX2* showed a hypermethylation pattern, whereas genes, such as *IL5RA*, *IL6*, and *IRF2*, showed lower level of methylation in comparison to LR risk carriers (Fig. 1a).

In addition, we observed significantly higher levels of methylation in genes including *STX8*, *ITGA9* and *ITPK1*, *INSR* in LR carrier compared to HRs (Fig. 1a).

Network and pathway enrichment analyses revealed that the DMGs were significantly enriched in pathways related to the nervous system and neurodegenerative diseases, including the synaptic vesicle cycle, GABAergic synapses, spinocerebellar ataxia, and Alzheimer's disease (Fig. 1b,c). We also observed a significant enrichment related to viral pathways, including hepatitis C and human papillomavirus infection (Fig. 1b,c).

In the LR carriers, we specifically observed enrichment in Type 2 diabetes (T2D)‐related pathways, such as insulin resistance, insulin secretion, and various signaling cascades, especially among the genes hypomethylated. On the other hand, the genes hypomethylated in the HR group were enriched for antigen presentation, autoimmune, inflammatory, and immune‐related pathways (Fig. 1c, Fig. S5).

**Methylation patterns in T1D developers compared to healthy controls** to extend our findings, we compared methylation patterns in T1D progressors with HR or LR HLA alleles using healthy controls carrying LR risk HLA alleles as a baseline reference. Comparison of DNA methylation profiles confirmed that T1D individuals with different HLA risk levels display distinct methylation signatures in cord blood DNA compared to healthy controls.

**Fig. 2:**
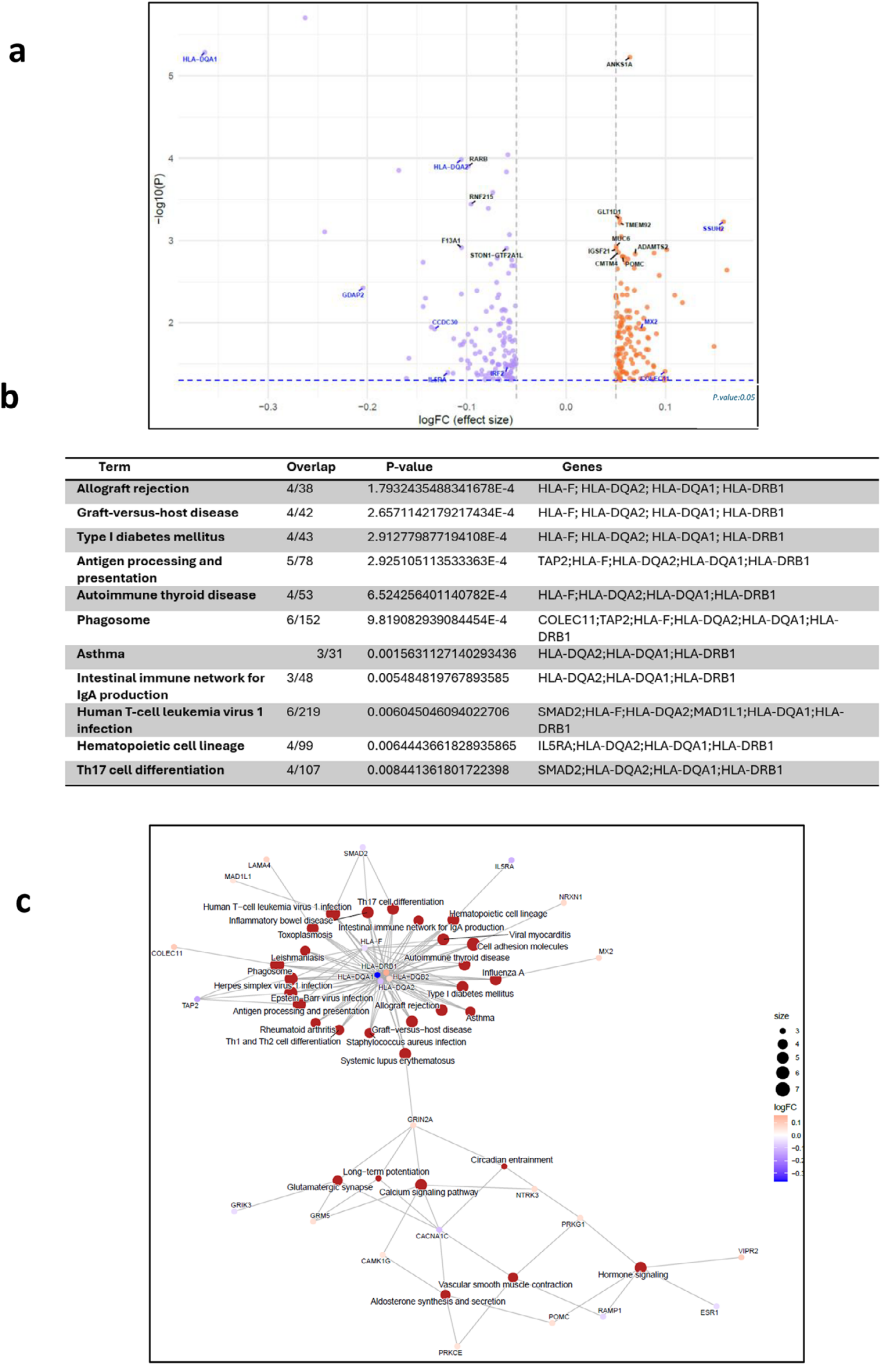
Methylation analysis comparing individuals with T1D carrying HR human leukocyte antigen (HLA) alleles to healthy controls. (a) Volcano plot illustrating the DMGs between the two groups (log FC: blue: <−0.05, red: >0.05). (b) Kyoto Encyclopedia of Genes and Genomes (KEGG) pathway enrichment analysis of DMGs (top 10 pathways enriched from the DMGs, p < 0.05). (c) Network visualization of enriched pathways and associated genes from DMC analysis. The network visualizes the relationships between enriched biological pathways and their associated genes. Each colored node (circle) represents either a pathway (in red) or a gene, with the gene nodes color‐coded according to their log fold change (log FC) values. The log FC scale indicates methylation levels. Edges (lines) indicate which genes are involved in which pathways, illustrating that individual genes can contribute to multiple biological processes. DMGs, differentially methylated genes; HR, high‐risk; LR, low risk; T1D, Type 1 diabetes.

In the HR HLA group, HLA genes were among the top differentially methylated CpGs (Fig. 2). In this comparison we detected hypomethylation of *HLA‐DQA1* and *HLA‐DQA2* in HR carriers compared to healthy controls. In addition, *IRF2* and *IL5RA* were hypomethylated, and *COLEC11* and *MX2* were hypermethylated in HR carriers compared to healthy controls (Fig. 2a).

Pathway enrichment analysis for DMGs indicated strong enrichment for immune and autoimmune pathways in T1D progressors with HR alleles. Gene network analysis revealed that HLA genes play the central role in these pathways (Fig. 2b,c).

**Fig. 3:**
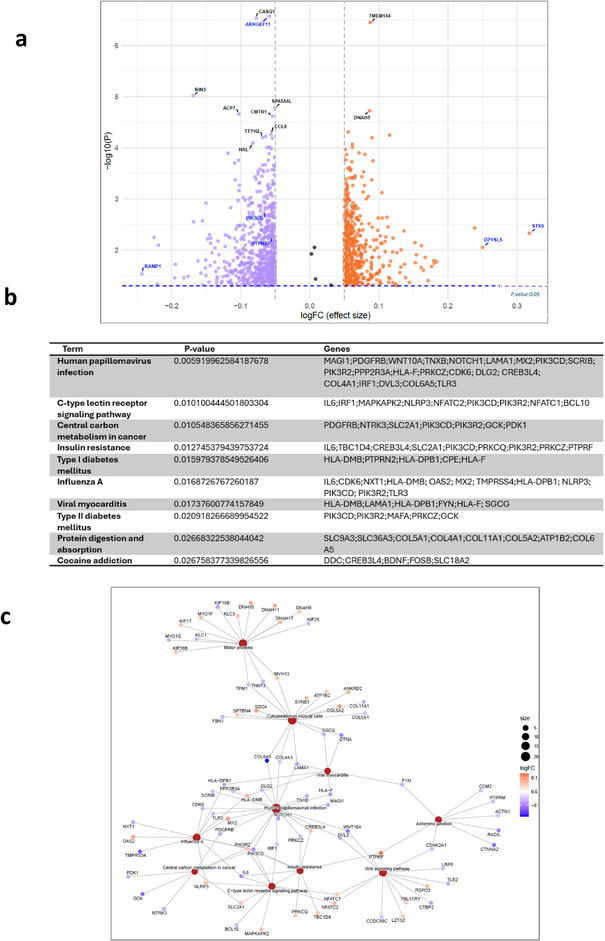
Methylation analysis comparing individuals with T1D carrying LR human leukocyte antigen (HLA) alleles to healthy controls. (a) Volcano plot illustrating the DMGs between the two groups (log FC: blue: <−0.05, red: >0.05). (b) Kyoto Encyclopedia of Genes and Genomes (KEGG) pathway enrichment analysis of DMGs (top 10 pathways enriched from the DMGs, p < 0.05). (c) Network visualization of enriched pathways and associated genes from DMC analysis. The network visualizes the relationships between enriched biological pathways and their associated genes. Each colored node (circle) represents either a pathway (in red) or a gene, with the gene nodes color‐coded according to their log fold change (log FC) values. The log FC scale indicates methylation levels. Edges (lines) indicate which genes are involved in which pathways, illustrating that individual genes can contribute to multiple biological processes. DMGs, differentially methylated genes; HR, high‐risk; LR, low risk; T1D, Type 1 diabetes.

In contrast, T1D progressors with LR risk alleles showed minimal involvement of HLA genes and reduced activation of immune pathways. We detected significant and strong hypermethylation in *STX8* and *DPYSL5* in LR alleles carriers compared to healthy controls. Furthermore, the methylation level of *ARHGEF11*, *CASQ1, PIK3CD*, and *PTN22* was reduced in this group compared to healthy controls (Fig. 3a).

The DMGs revealed in this group demonstrated predominant enrichment in metabolic pathways (including insulin resistance), cellular signaling cascades, neurogenic processes, and mechanisms associated with T2D (Fig. 3b,c). Figs. S3 and S4 present the genomic distribution of CpGs associated with the top DMGs in comparisons of T1D with HR alleles versus healthy controls and T1D with LR alleles versus healthy controls, respectively.

### Enrichment pathway analysis highlights autoimmune signatures just among HR HLA group

To investigate the epigenetic relationship between T1D and NT1D individuals with HR alleles compared to LR carriers and to examine the role of HLA risk alleles in the disease mechanism, we conducted separate analyses to identify DMCs between NT1D individuals and healthy controls using cord blood DNA. We found that HR carriers, regardless of whether they developed T1D or NT1D, shared a highly similar methylation pattern (Fig. S6).

**Fig. 4:**
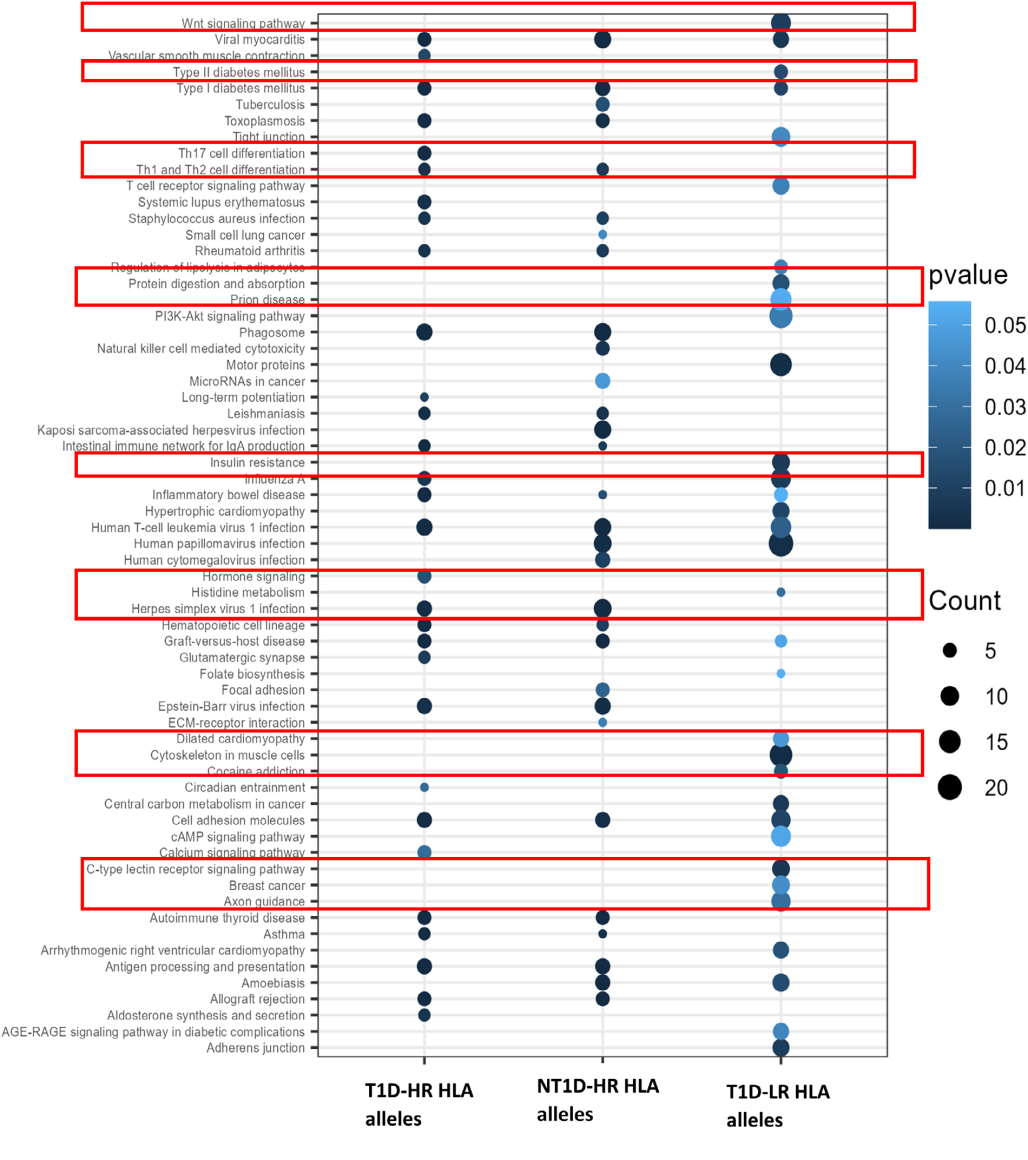
Enrichment pathways for the differentially methylated genes (DMGs) discovered from different comparisons. Pathway enrichment analysis demonstrates that individuals with HR human leukocyte antigen (HLA) alleles (both T1D and NT1D) show similar epigenetic patterns enriched for autoimmune mechanisms, while T1D individuals with LR HLA alleles exhibit metabolic pathway enrichment characteristic of Type 2 diabetes. HR, high‐risk; LR, low risk; NT1D, other autoimmune diseases except T1D; T1D, Type 1 diabetes.

We then compared the enrichment pathway patterns of these DMCs with those found between T1D patients carrying HR or LR alleles and healthy controls. The result further demonstrated that both T1D and NT1D individuals with HR HLA alleles showed similar enrichment patterns and were enriched for autoimmune mechanisms and immune‐related pathways. In contrast, T1D individuals with LR HLA alleles exhibited a distinct pattern, being enriched in metabolic and signaling pathways, resembling patterns more characteristic of T2D (Fig. 4) (Table 1).

**Table 1:** Key differentially methylated genes in cord blood DNA of Type 1 diabetes (T1D) cases with different human leukocyte antigen (HLA) risk alleles.

Comparison group	Gene	Biological context	Established function/relevance	Ref.
**T1D‐HR vs. T1D‐LR**	**HLA‐DQA1**	Antigen presentation (MHC Class II)	HLA Class II α‐chain; presents antigen to CD4+ T cells; major T1D risk via DQ2/DQ8	[42]
	**HLA‐DQA2**	Antigen presentation (MHC Class II)	Supports DQ heterodimer and susceptibility to autoimmunity	[43]
	**IL5RA**	Cytokine signaling	IL‐5 receptor α; eosinophil activation; Th2 inflammation	[44]
	**IL6**	Cytokine signaling/JAK–STAT signaling	Proinflammatory cytokine contributing to immune dysregulation and metabolic complications in T1D/T2D	[45, 46]
	**IRF2**	A transcription factor from interferon regulatory factor (IRF) family	A negative regulator of IFN‐α/β signaling, regulates antiviral and immune gene expression	[47]
	**ARHGEF11**	Rho GTPase signaling	Linked to T2D risk, insulin resistance; metabolic regulation	[48, 49]
	**PTPN22**	T‑cell signaling/autoimmunity	Negative regulator of T‐cell activation; T1D/T2D susceptibility	[50, 51]
	**PIK3CD**	PI3K signaling pathway, insulin signaling pathways	PI3K p110δ; controls lymphocyte activation and insulin‐related pathways	[52]
	**MX2**	Type I interferon response	IFN‐induced antiviral protein; protects β‐cells from enterovirus	[53, 54]
	**STX8**	Beta‐cell function/insulin Secretion	Regulates insulin granule turnover and endosomal trafficking	[55]
	**ITGA9**	Inflammatory signaling/cell adhesion	Integrin involved in inflammatory insulin resistance; osteopontin receptor	[69, 70]
	**ITPK1**	Insulin signaling	Regulates AKT1 activation, β‐cell mass, and secretion	[56, 57]
	**INSR**	Insulin signaling	Core insulin receptor; essential for glucose uptake	[58]
**T1D‐HR vs. Healthy‐LR**	**HLA‐DQA1**	Antigen presentation (MHC Class II)	HLA class II α‐chain; presents antigen to CD4+ T cells; major T1D risk via DQ2/DQ8	[42]
	**HLA‐DQA2**	Antigen presentation (MHC Class II)	Supports DQ heterodimer and susceptibility to autoimmunity	[43]
	**IRF2**	A transcription factor from interferon regulatory factor (IRF) family	A negative regulator of IFN‐α/β signaling, regulates antiviral and immune gene expression	[47]
	**IL5RA**	Cytokine signaling	IL‐5 receptor α; eosinophil activation; Th2 inflammation	[44]
	**MX2**	Type I interferon response	IFN‐induced antiviral protein; protects β‐cells from enterovirus	[53, 54]
**T1D‐LR vs. Healthy‐LR**	**STX8**	Beta‐cell function/Insulin secretion	Regulates insulin granule turnover and endosomal trafficking	[55]
	**DPYSL5**	Axon guidance/Neurodevelopment	CRMP5; neuronal development and axonal patterning	[59]
	**ARHGEF11**	Rho GTPase signaling	Linked to T2D risk, insulin resistance; metabolic regulation	[48, 49]
	**CASQ1**	Calcium handling/Ca^2+^ homeostasis	Regulates Ca^2+^ storage/release; influences β‐cell excitability	[60, 61]
	**PIK3CD**	PI3K signaling pathway, insulin signaling pathways	PI3K p110δ; controls lymphocyte activation and insulin‐related pathways	[52]

Abbreviations: HR, high risk; T2D, Type 2 diabetes.

## Discussion

In our study, we compared the methylation profiles of cord blood DNA from individuals who later developed T1D, focusing on differences associated with their HLA genotypes. To investigate potential links between these methylation patterns and autoimmune diseases more broadly, we also compared our findings to methylation profiles from individuals with autoimmune diseases other than T1D. Our results show that HLA genotype is associated with distinct early‐life DNA methylation patterns, which may influence molecular pathways involved in development/triggering of T1D. We observed that individuals with HR HLA genotypes for T1D share methylation signatures and affected pathways similar to those seen in NT1D autoimmune diseases, whereas individuals with LR HLA genotypes exhibited distinct methylation patterns, suggesting different underlying mechanisms in disease development.

T1D is regarded as an autoimmune disease, which is characterized by development of several different autoantibodies, which tend to appear already within 1–2 years of age. Gradually those with multiple autoantibodies, a hallmark of Stage 1 T1D, develop glucose intolerance, which is Stage 2 of T1D, and then it usually passes to Stage 3 within 1–2 years, which is clinically manifest diabetes with increased blood glucose. The etiology is unknown [1] but most probably dependent on many different factors, probably starting already in fetal life. Thus, already in cord blood, the protein pattern shows an inflammatory pattern supported by metabolomic findings in cord blood. Harmful agents such as per‐ and polyfluoroalkyl substances seem to be increased in individuals who later develop autoimmune diseases [62], and it is known for decades that enterovirus infections during pregnancy increase the risk for T1D in the child later [63]. There are several large birth cohort studies trying to elucidate etiology of T1D, the largest being the TEDDY study [64]. However, TEDDY, as well as the DIPP study in Finland, BabyDiab in Germany, DAISY in Denver, USA, and the recently started ENDIA study in Australia, all include just HR individuals with HR HLA alleles and/or T1D in the family. The high genetic risk may be so dominating that environmental factors are overshadowed and not seen, as HLA genes may influence both factors of importance for the development of the immune system, such as the gut microbiome [19] and the immune system per se.

As environmental factors may cause activation and inactivation of gene activity, epigenetic studies may be a way forward. Such studies have been done with inconclusive findings in cord blood. Thus, Lahesma and coworkers [10, 65] studied epigenetics in the cord blood of DIPP‐ individuals and found no differences in the umbilical cord blood methylation patterns between the cases and controls at a false discovery rate <0.05. They therefore concluded that differences between children who progress to T1D and those who remain healthy throughout childhood are not yet present in the perinatal DNA methylome. However, a limitation may be that they compared cases and controls with the same HLA types, taken from the DIPP material [10].

A separate study by Cepek et al. examined DNA methylation and mRNA expression of *HLA‐DQA1* alleles in T1D. They reported that although methylation at the *HLA‐DQA1* promoter was similar overall between T1D patients and controls, methylation was allele‐specific (with DQA1*02:01exhibiting the highest and DQA1*05:01 the lowest methylation). It should be noted that their study analyzed a mixed cohort of T1D individuals with various HLA backgrounds [66].

A German study [67] found marked differences in the methylation status of CpG sites within the MHC genes *cis*‐metQTLs between carriers of T1D with risk haplotypes HLA‐DRB1*03:01‐DQA1*05:01‐DQB1*02:01 (DR3‐DQ2) and HLA‐DRB104:01‐DQA103:01‐DQB103:02(DR4‐DQ8).

These differences were found in children and adults and were accompanied by reduced HLA‐DR protein expression in immune cells with the *HLA‐DR3‐DQ2* haplotype, which supports our idea to study epigenetic changes of individuals who develop T1D later with or without HLA risk, as our studies are based on the ABIS birth cohort, which is unique in following a general population.

In our study, we found that HLA genes ranked among the top differentially methylated CpGs in HR HLA allele carriers, which were not observed in LR group. Specifically, *HLA‐DRB1* was hypermethylated, whereas *HLA‐DQA1* and *HLA‐DQA2* were hypomethylated in HR group. The pattern of HLA gene methylation in HR T1D cases closely resembled the pattern we observed in NT1D individuals with HR HLA genotypes.

The hypomethylation pattern of HLA genes is reported in other studies related to autoimmune diseases and is believed to allow higher gene expression of HLA genes on the surface of immune cells, possibly contributing to T1D risk in HR carriers [66, 67]. Our finding is in line with established mechanisms by which HLA‐DR/DQ alleles influence the initiation and progression of T‐cell–mediated autoimmunity that targets pancreatic β‐cells, especially in genetically susceptible individual [2].

We observed hypermethylation in *HLA‐DRB1* in HR allele carriers. A number of previous evidence reports reduced methylation of *HLA‐DRB1* in autoimmune diseases such as multiple sclerosis [68], psoriasis [69], and systemic lupus erythematosus (SLE) [70]. However, the effect of methylation on *HLA‐DRB1* expression and autoimmune risk is allele‐specific and complex and can be strongly driven by genetic variation (allele‐specific methylation).

In our study, the methylation pattern in T1D progressors carrying LR HLA alleles was different and less involved HLA genes. This may suggest involvement of different molecular mechanisms involved in beta cell destruction and insulin‐related dysfunctions in LR carriers.

For example, we observed significant hypomethylation of Rho Guanine Nucleotide Exchange Factor 11 (*ARHGEF11*) gene in LR risk carriers. The genetic variants in *ARHGEF11* are highly associated with increased risk of T2D and altered glucose metabolism across multiple populations. This gene is linked to the increased measures of insulin resistance such as, fasting plasma glucose (FPG) and fasting insulin [48]. Furthermore, a recent study reported *ARHGEF11* is significantly hypomethylated in the cord blood in macrosomic infants. In that study, altered DNA methylation levels of *ARHGEF11* were negatively correlated with glucose levels and neonatal birth weight [49]. Hypomethylation of this gene and its consequent change in its expression in LR risk carriers may increase individual susceptibility for starting mechanisms that cause disease in the future.

Calsequestrin (*CASQ1*) is involved in intracellular storage and release of calcium, a process that has been shown to mediate glucose transport. The polymorphism of this gene is associated with altered T2D susceptibility in different populations through effects on gene expression regulation [60, 61]. In our study, we observed hypomethylation of this gene in LR carriers, which may affect the *CASQ1* regulation of glucose metabolism through epigenetic and impact metabolic pathways leading to beta cell dysfunction.

We also detected a strong hypermethylation of *STX8*, *ITGA9*, and *ITPK1* in LR risk carriers compared to HR carriers. Syntaxin 8 (*STX8*) regulates insulin granule turnover and secretion in pancreatic beta cells, and it is required for its endosomal trafficking. It has been reported that expression of *SXT8* in visceral adipose tissue is increased in obese patients with T2D and is linked to insulin resistance and inflammation [55]. Regarding the importance of *STX8* in insulin homeostasis, dysfunction in this process can disturb insulin levels and is implicated as a diabetes risk mechanism [71]. Hypermethylation of *STX8* in cord blood DNA from LR carriers may signal future susceptibility to beta cell dysfunction via a metabolically related pathway.

Interestingly, proteomic analyses also revealed differences in the serum levels of STX8 and members of the ARHGEF gene family in low‐risk HLA carriers compared with controls (Fig. S7).

Integrin Alpha‐9 (*ITGA9*) is implicated in inflammatory insulin resistance within muscle and adipose tissues and mediates cell adhesion and immune regulation. Recent evidence shows that *ITGA9* functions as a receptor for osteopontin released by endothelial cells during metabolic stress [72]. Binding osteopontin to *ITGA9* on macrophages triggers inflammation, directly impairing insulin‐stimulated glucose uptake in muscle and thereby intensifying insulin resistance. *ITGA9*‐associated signaling complexes in adipocytes further influence the inflammatory microenvironment, modulating tissue insulin sensitivity and diabetes risk [73, 74].

Inositol‐tetrakisphosphate 1‐kinase (*ITPK1*) is a key modulator of insulin signaling via AKT1 phosphorylation and beta cell insulin production. *ITPK1* plays a critical role in inositol phosphate metabolism. Compromised *ITPK1* impaired glucose tolerance, diminished beta cell mass and insulin secretion, and increased vulnerability to diet‐induced diabetes and insulin resistance in mouse models [56, 57].

Interestingly, we observed slight hypomethylation of *GAD2* in HR carriers compared to LRs, which is a major T1D autoantigen [75]. *GAD2* encodes the enzyme glutamic acid decarboxylase 65 (*GAD65*), which catalyzes the conversion of glutamate to gamma‐aminobutyric acid (GABA) and is critically involved in both the brain and insulin‐producing pancreatic beta cells [75]. Different methylation status of this gene in HR carriers may potentially increase the susceptibility of these individuals to develop autoimmune T1D later, whereas further research is needed to support this link.

Myxovirus resistance 2 (Mx2) expression is strongly associated with autoimmune diseases, including SLE, through innate immune mechanisms and interferon (IFN) pathways [54]. T1D is thought to be triggered by environmental factors, with enteroviruses identified as prominent stimulators of the disease. MX2 functions downstream of Type I interferon signaling, a pathway critical to T1D pathogenesis. Increased interferon activity has been linked to elevated MX2 expression in both pancreatic islets and peripheral immune cells in diabetes models and patients [53, 54].

Interestingly, we observed the pattern of methylation in *MX2* gene was affected by HLA background. Although it showed a hypomethylated pattern in LR carriers, it was hypermethylated in the HR ones. Hypermethylation of *MX2* and following decreased expression may make the beta cells more susceptible to virus‐triggered cell damage and make these cells vulnerable to viral infections during inflammatory (high IFN) periods, possibly increasing the risk of viral‐driven beta cell destruction and promoting autoimmune diabetes. Damaged or dying beta cells are more likely to release autoantigens, potentiating or accelerating autoimmune destruction.

*PIK3CD*, PTPN22, and *IL6* are other genes with different methylation patterns in T1D developer of varying HLA background and are also known for important role in T2D development, insulin signaling, and inflammation [76].

Our analysis also revealed significant hypermethylation of Dihydropyrimidinase‐like 5 (*DPYSL5/CRMP5*) in the cord blood DNA of T1D‐developing individuals with low to neutral risk HLA alleles compared to healthy controls, whereas this was not detected when comparing LR to HR T1Ds. *DPYSL5* is primarily associated with neurodevelopmental processes [59].

Pathway enrichment analysis of the DMG in these two groups further confirmed the heterogeneity of mechanisms in T1D development regarding HLA background. DMG in HR carriers in both individuals with T1D or other autoimmune diseases was mostly enriched in immune‐related mechanisms and autoimmune disease. This was significantly different with the pattern of enrichment in T1D developers that carry an LR HLA. DMG in the latter group were enriched with metabolic and signaling pathways and less related to immune pathways.

In addition, our proteomic analyses revealed differences between T1D‐HR and T1D‐LR individuals in serum levels of immune‐related as well as metabolic‐ and stress‐related proteins measured at birth (Figs. S8 and S9). Immune‐related proteins were associated with pathways involved in immune signaling and regulation, whereas metabolic‐ and stress‐related proteins were linked to glucose and lipid metabolism, endoplasmic reticulum stress responses, and cellular survival. Together, these findings support early‐life biological heterogeneity across HLA‐defined risk groups in individuals who later develop T1D.

Although our pathway enrichment analysis of DMGs robustly identifies biologically relevant immune and autoimmune pathways in HR HLA carriers versus metabolic/insulin‐signaling pathways in low‐to‐neutral‐risk carriers, interpreting the direction of pathway regulation requires considering the genomic context of CpGs (e.g., promoter, enhancer, gene body, and CpG island/shores). Most associated CpGs were located within gene bodies, where methylation often is linked to active transcription. However, we also observed differentially methylated CpGs upstream of the transcriptional start sites, where methylation is more associated with transcriptional repression [77]. Thus, the regulatory effect of hyper‐ and hypomethylation is context‐dependent and cannot be inferred solely from the direction of methylation change. Our EWAS, therefore, emphasizes pathway associations to reveal T1D mechanistic heterogeneity across HLA risk groups, rather than inferring direct regulatory impacts.

In our study, functional relevance of gene‐level epigenetic differences was further supported by proteomic analyses of serum collected at birth, demonstrating cross‐layer consistency between differential DNA methylation and downstream protein level (Fig. S7).

Integrating methylation data with transcriptomic or proteomic (e.g., Olink) profiles would strengthen regulatory inferences linking methylation to downstream gene‐expression changes. Future work incorporating these advanced validation approaches will further deepen our understanding of T1D mechanistic heterogeneity.

## Conclusion

Our study shows several distinct patterns for pathway enrichment between individuals with HR HLA who later develop T1D and those who develop T1D in spite of LR HLA alleles. Those with HR HLA alleles showed similar epigenetic patterns, as expected, as individuals who developed other autoimmune diseases, with traits related to changes in immune reactions. In addition, we found epigenetic changes indicating that virus infections during pregnancy may play a role.

The individuals who later developed T1D without HR alleles have other epigenetic patterns, not least pathways associated with insulin resistance and what is seen in individuals with T2D. Several facts suggest that beta cell stress is of great importance of the development for T1D [18]. In addition, this may be of special importance in individuals without high genetic risk of getting T1D.

It seems that a genetic and epigenetic background, established at birth, may influence the susceptibility of the individual in driving an autoimmune or non‐autoimmune mechanism in developing T1D later in life. Prenatal factors such as the maternal lifestyle and infection history can modify these genetic and epigenetic predispositions, further impacting disease risk.

## Author contributions

Conceptualization: Johnny Ludvigsson. Data curation: Shamila D. Alipoor and Johnny Ludvigsson. Formal analysis: Shamila D. Alipoor and Julia Åkesson. Funding acquisition: Johnny Ludvigsson. Investigation methodology: Shamila D. Alipoor, Maria Lerm, Julia Åkesson, Mika Gustafsson, and Thomas Hillerton. Project administration: Johnny Ludvigsson and Maria Lerm. Resources software: Johnny Ludvigsson. Supervision: Johnny Ludvigsson and Maria Lerm. Validation: Shamila D. Alipoor, Johnny Ludvigsson, and Maria Lerm. Visualization: Shamila D. Alipoor. Writing—original draft writing: Shamila D. Alipoor. Review and editing: Shamila D. Alipoor, Angelica Ahrens, Julia Åkesson, Thomas Hillerton, Mika Gustafsson, Maria Lerm, and Johnny Ludvigsson.

## Conflict of interest statement

The authors declare no conflicts of interest.

## Disclosure

The manuscript has been handled by an external editor: Ulf Smith, Professor, Department of Molecular and Clinical Medicine, Institute of Medicine, University of Gothenburg, Sweden.

## Funding information

ABIS was supported by Barndiabetesfonden (Swedish Child Diabetes Foundation); Swedish Research Council Grant Numbers: K2005‐72X‐11242‐11A and K2008‐69X‐20826‐01‐4, K2008‐69X‐20826‐01‐4; Medical Research Council of Southeast Sweden (FORSS); JDRF Wallenberg Foundation, Grant Number: K 98‐99D‐12813‐01A; ALF and LFoU grants from Region Östergötland and Linköping University, Sweden, and the Joanna Cocozza Foundation.

## Supporting information




**Fig. S1**: joim70077‐sup‐0001‐SuppMat.png.

**Fig. S2**: joim70077‐sup‐0002‐SuppMat.png.

**Fig. S3**: joim70077‐sup‐0003‐SuppMat.png.

**Fig. S4**: joim70077‐sup‐0004‐SuppMat.png.

**Fig. S5**: joim70077‐sup‐0005‐SuppMat.png.

**Fig. S6**: joim70077‐sup‐0006‐SuppMat.png.

**Fig. S7**: joim70077‐sup‐0007‐SuppMat.jpg.

**Fig. S8**: joim70077‐sup‐0008‐SuppMat.png.

**Fig. S9**: joim70077‐sup‐0009‐SuppMat.png.

**Table S1**: joim70077‐sup‐0010‐SuppMat.pdf.

## Data Availability

The data were curated in compliance with the necessary ethical human subjects’ protections for our ABIS participants. Researchers who are interested in obtaining additional information or data that underline this paper should contact the lead authors. For any data that are not currently available via open access, researchers may need to develop a formal collaboration with the study group.
