# Supplementary figures and images for: Epigenetic analyses suggest different pathways during pregnancy for development of type 1 diabetes in children with high versus low‐neutral human leukocyte antigen‐risk
Source: J Intern Med. 2026 Feb 25;299(5):570–86. doi: 10.1111/joim.70077 (PMC13061103; doi:10.1111/joim.70077)

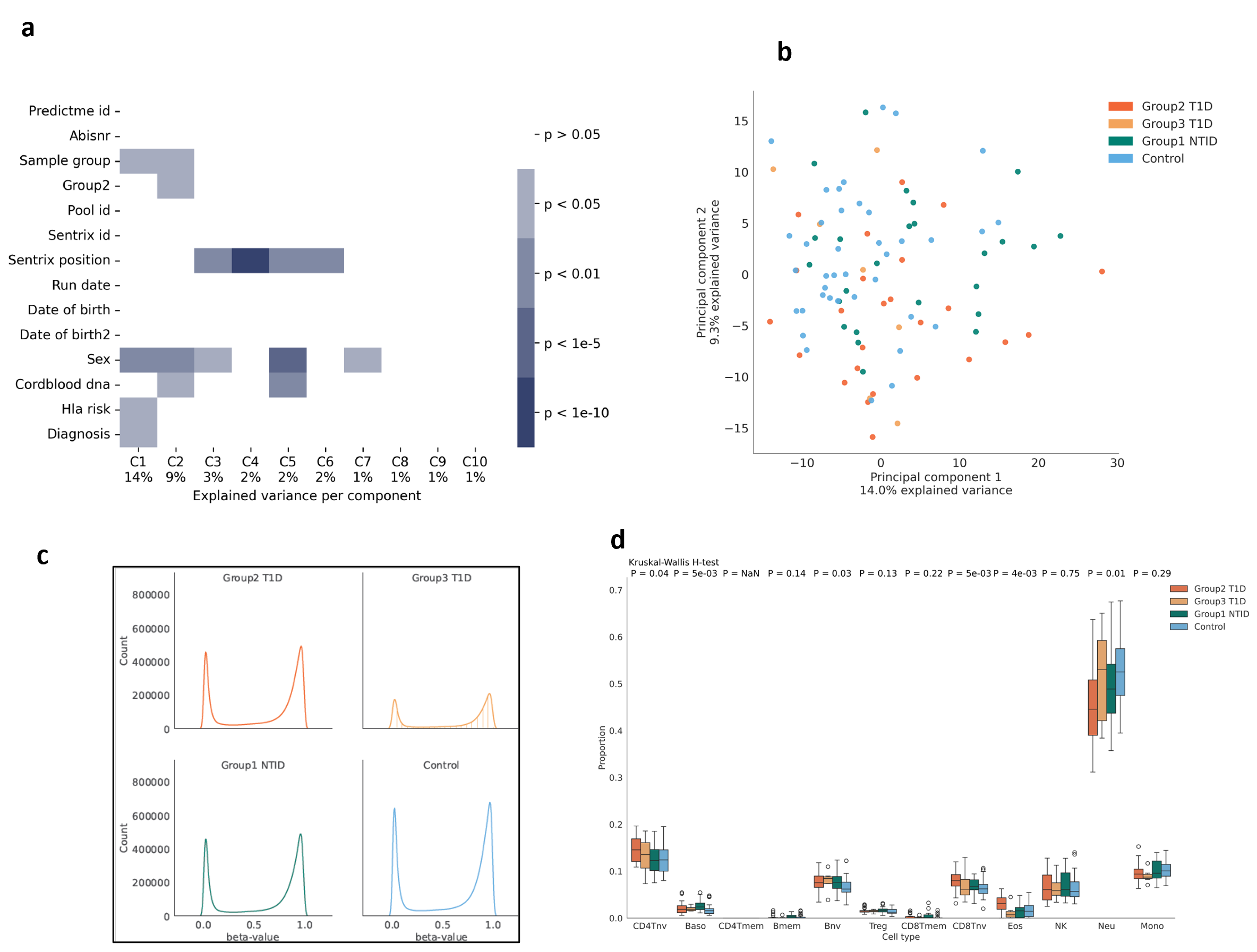

Supplement: Supplementary file 1 — Fig. S1: joim70077‐sup‐0001‐SuppMat.png. [file JOIM-299-570-s002.png]

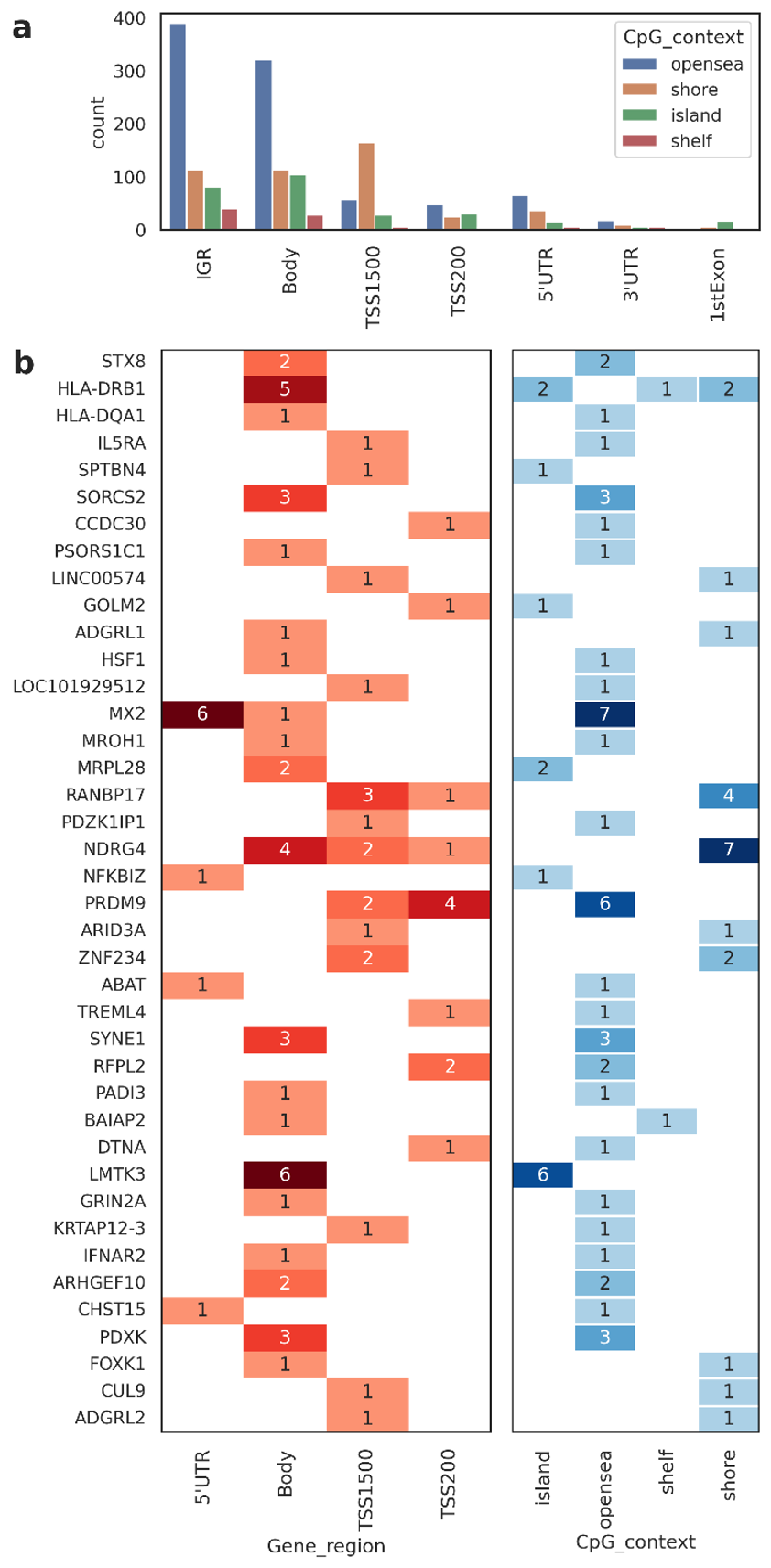

Supplement: Supplementary file 2 — Fig. S2: joim70077‐sup‐0002‐SuppMat.png. [file JOIM-299-570-s006.png]

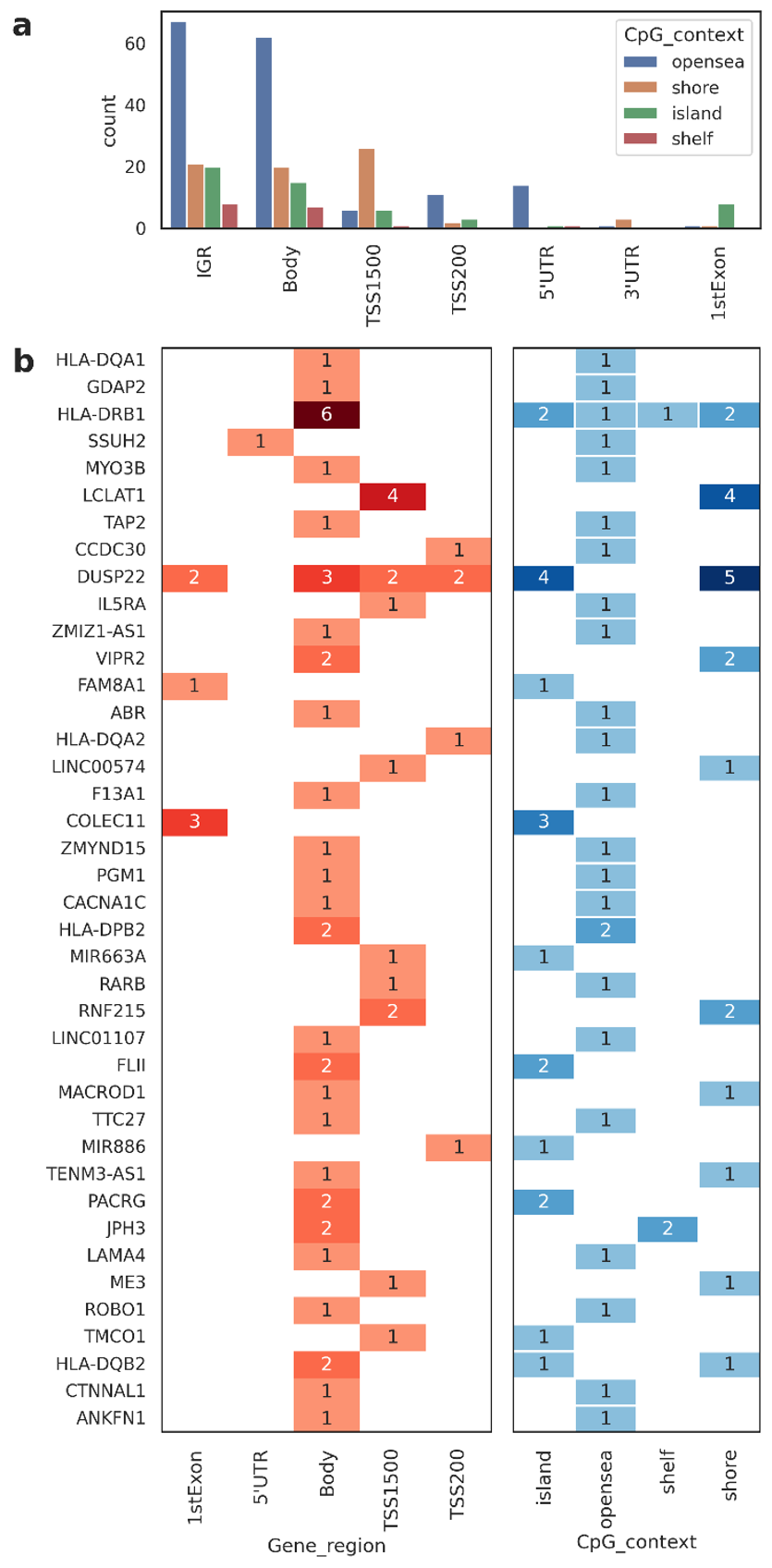

Supplement: Supplementary file 3 — Fig. S3: joim70077‐sup‐0003‐SuppMat.png. [file JOIM-299-570-s008.png]

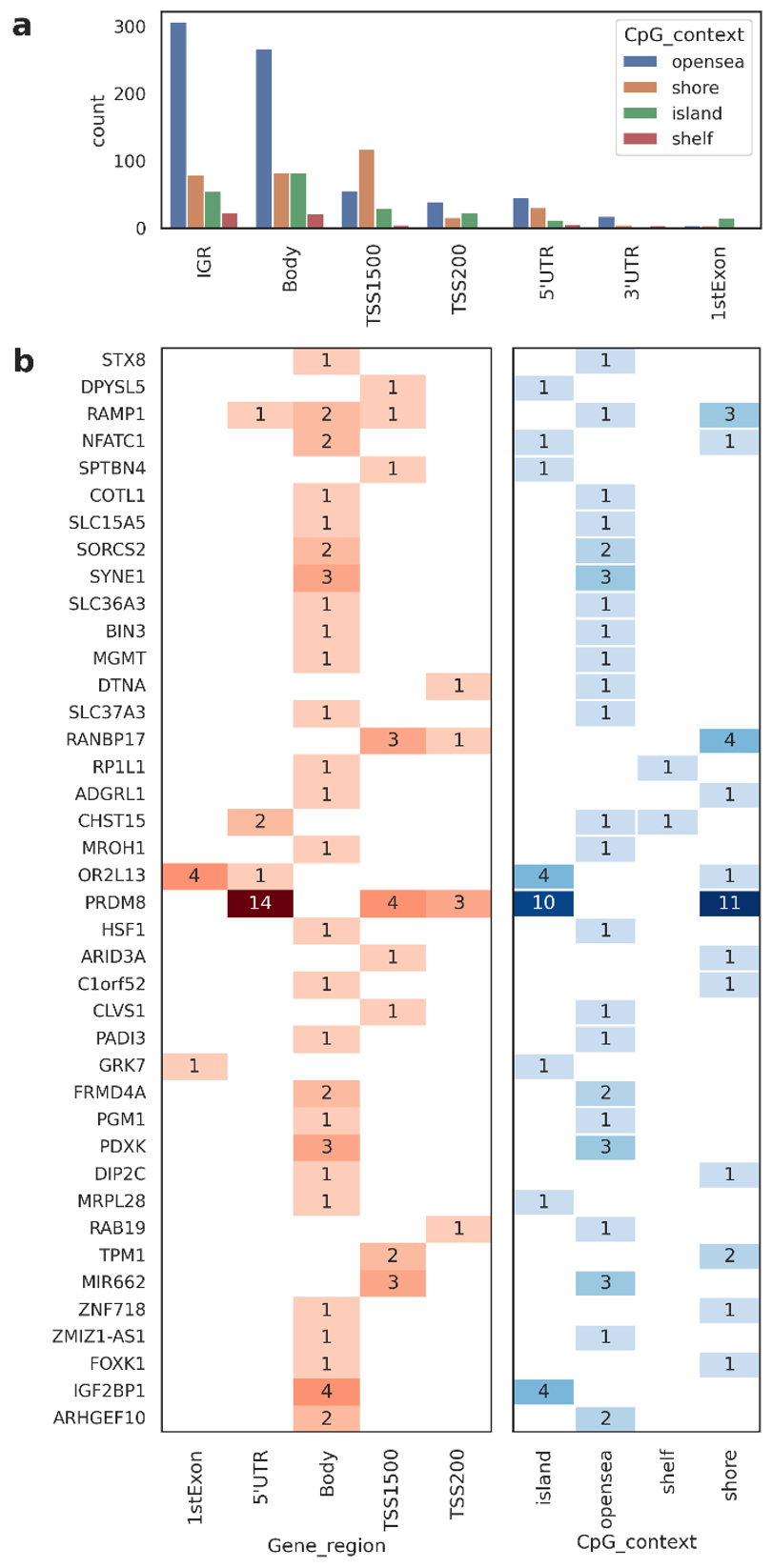

Supplement: Supplementary file 4 — Fig. S4: joim70077‐sup‐0004‐SuppMat.png. [file JOIM-299-570-s003.png]

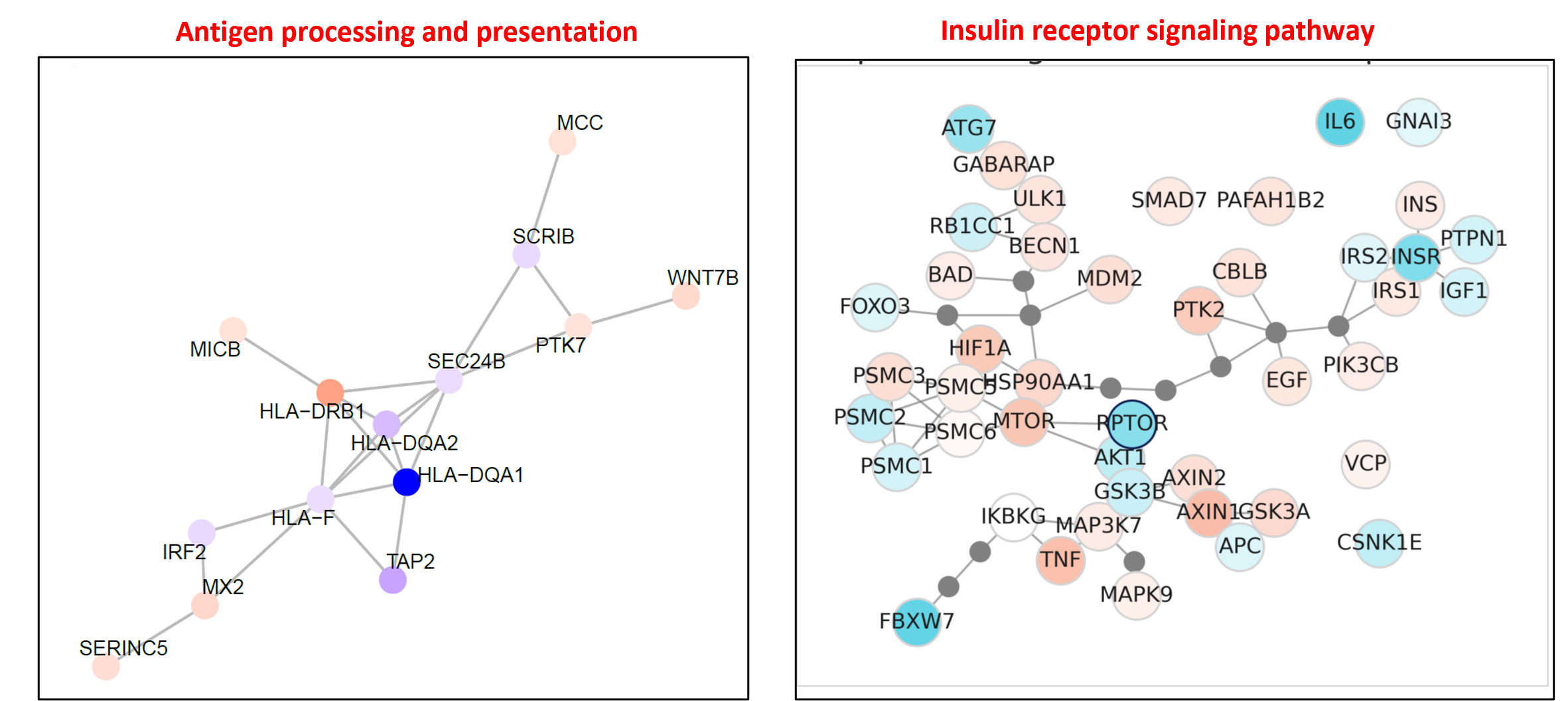

Supplement: Supplementary file 5 — Fig. S5: joim70077‐sup‐0005‐SuppMat.png. [file JOIM-299-570-s004.png]

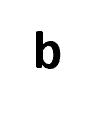

Supplement: Supplementary file 6 — Fig. S6: joim70077‐sup‐0006‐SuppMat.png. [file JOIM-299-570-s001.png]

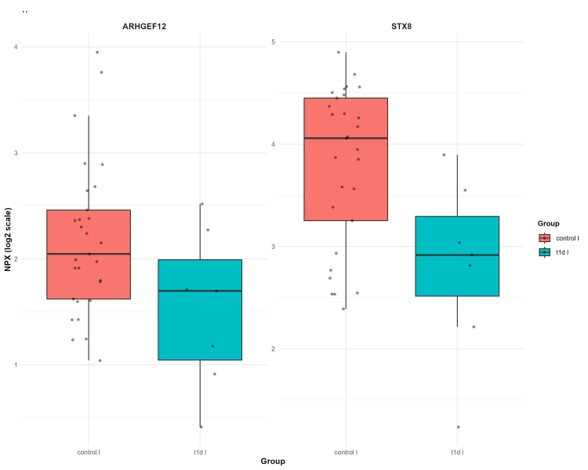

Supplement: Supplementary file 7 — Fig. S7: joim70077‐sup‐0007‐SuppMat.jpg. [file JOIM-299-570-s009.jpg]

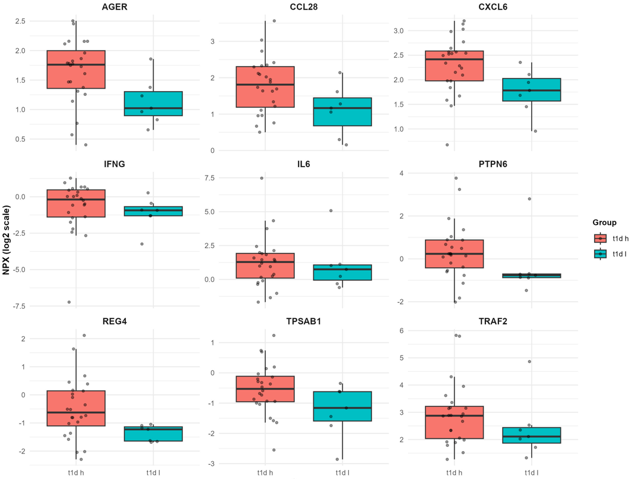

Supplement: Supplementary file 8 — Fig. S8: joim70077‐sup‐0008‐SuppMat.png. [file JOIM-299-570-s007.png]

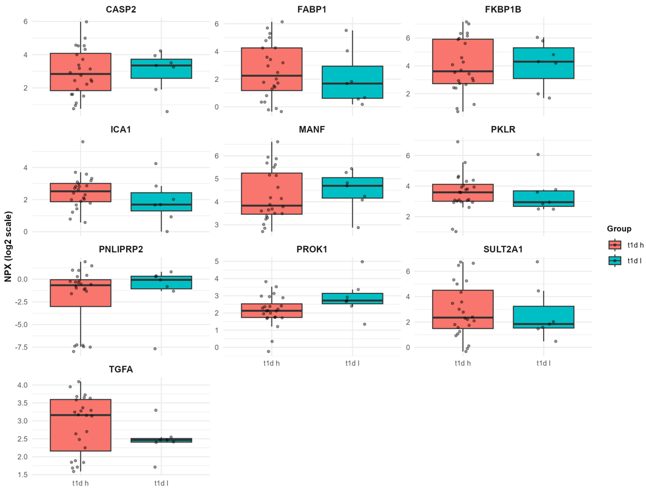

Supplement: Supplementary file 9 — Fig. S9: joim70077‐sup‐0009‐SuppMat.png. [file JOIM-299-570-s005.png]
