# Supplementary material for: Epigenetic analyses suggest different pathways during pregnancy for development of type 1 diabetes in children with high versus low‐neutral human leukocyte antigen‐risk
Source: J Intern Med. 2026 Feb 25;299(5):570–86. doi: 10.1111/joim.70077 (PMC13061103; doi:10.1111/joim.70077)
Supplement: Supplementary file 10 — Table S1: joim70077‐sup‐0010‐SuppMat.pdf. [file JOIM-299-570-s010.pdf]

LR/Healthy

Region/ count	Count(total DMCs:306)	Cpg Contex	Percentage(%)
IGR(intergenomic region)	464(34,4%)	Island	0,220913108
		Island	6,111929308
		Open sea	19,66126657
		Shelf	1,620029455
Body (of the gene)	N=455(33,5%)	Shore	6,111929308
		Island	4,050073638
		Open sea	22,60677467
		Shelf	1,693667158
Core promoter	34,17%	Shore	5,817378498
		Island	3,902798233
	Core promoter	Open sea	6,995581738
	291(21%)	Shelf	0,441826215
		Shore	9,793814433

HR/Healthy

Region	Count(total DMCs:306)	Cpg Contex	Percentage(%)
IGR(intergenomic region)	117(38%)	Open sea	56,41026
		Island	17,09402
		Shores	17,94872
		Shelf	6,837607
Body (of the gene)	105(34%)	Open sea	57,14286
		Island	18,09524
		Shores	18,09524
		Shelf	6,666667
Core promoter	56(18%)	Open sea	48%
		Island	16%
		Shores	31%
		Shelf	3%
